# Analysis of *cis* Elements in the *mp50* mRNA of *Trichomonas vaginalis*

**DOI:** 10.3390/microorganisms14081755

**Published:** 2026-08-10

**Authors:** José Luis Villalpando-Aguilar, Elisa Elvira Figueroa-Angulo, Luis Alberto Salazar-Pedreguera, Edgar Gerardo Castillo-Hernández, María Elizbeth Alvarez-Sánchez

**Affiliations:** 1 Laboratorio de Patogenesis Celular y Molecular Humana y Veterinaria, Posgrado en Ciencias Genomicas, Universidad Autonoma de la Ciudad de Mexico, San Lorenzo 290, Colonia del Valle, Delegación Benito Juárez México, Mexico City 03100, Mexico; villalpandojoseluis30@gmail.com (J.L.V.-A.); figueroaelisaelvira@gmail.com (E.E.F.-A.); luis.salazar@alumnos.uacm.edu.mx (L.A.S.-P.); edgar.castillo@estudiante.uacm.edu.mx (E.G.C.-H.); 2 Secretaria de Ciencias, Humanidades, Tecnologia e Innovacion (SECIHTI), Benito Juarez, Ciudad de Mexico 03940, Mexico

**Keywords:** *Trichomonas vaginalis*, metalloproteinase, biomarker, PAT-PCR, poly-A tail, zinc, post-transcriptional regulation

## Abstract

*Trichomonas vaginalis* is the protozoan parasite responsible for trichomoniasis, a prevalent non-viral sexually transmitted disease where high Zn^2+^ concentrations in the male prostate microenvironment function as a natural infection barrier. This study evaluates the post-transcriptional mechanisms governing the gene expression and mRNA stability of the 50 kDa metalloproteinase (TvMP50), an immunogenic biomarker for male trichomoniasis, under zinc stress. Using *T. vaginalis* female (CNCD147) and male (HGMN01) isolates, *mp50* expression was characterized through transcription inhibition assays with Actinomycin-D, 5′ RACE, 3′ RACE, poly (A) tail assays (PAT-PCR), and Mfold secondary structure modeling. Results demonstrated that while transcription consistently initiates at a 10-nucleotide sequence relative to the start codon, the presence of 1.6 mM Zn^2+^ significantly stabilizes the transcript, extending its experimental half-life from 30 to 47 min. Furthermore, PAT-PCR and sequencing confirmed that Zn^2+^ exposure alters poly (A) tract lengths, reaching up to ~700 nucleotides, and utilizes alternative polyadenylation and cleavage sites to form stable 3′ untranslated region stem-loop configurations. In conclusion, *mp50* overexpression under zinc stress is driven by a post-transcriptional mechanism where the synergistic effect of alternative processing signals and longer poly (A) tails enhances mRNA stability, optimizing parasite virulence during male urogenital colonization.

## 1. Introduction

*Trichomonas vaginalis* is the causal agent of trichomoniasis, the most frequent non-viral sexually transmitted infection (STI) in the world, accounting for roughly 156 million new annual cases among individuals aged 15 to 49, exceeding the combined worldwide annual prevalence incidence rates of other STIs [1,2]. Racial and social disparities, the number of sexual partners, and the epidemiologic association with other STIs are some of the risk factors that contribute to trichomoniasis prevalence. This extracellular flagellated protozoan parasite is transmitted directly through genital fluid interchange, colonizing the epithelium of the human urogenital tract (HUT). Although this parasite can survive in female and male urogenital microenvironments, female trichomoniasis is usually symptomatic and associated with reproductive morbidity [1,3]. At least 77% of male infections are asymptomatic and usually resolve on their own. The pathogen causes chronic prostatic inflammation and hyperplastic lesions that can contribute to prostate cancer (PC) susceptibility due to the secretion of pro-inflammatory cytokines [4,5,6,7]. The high Zn^2+^ concentrations found in the prostate microenvironment and prostatic fluids (ranging from 4.5 to 7 mM of Zn^2+^) allow for natural infection control, with 1.6 mM being the concentration described as the minimal trichomonacidal concentration [8,9,10]. The relationship between *T. vaginalis* infection and cancer was previously reported in epidemiological and meta-analysis studies [11,12].

The *T. vaginalis* genome is approximately 160 Mb in size, distributed across six chromosomes and consisting of 65% repetitive sequences corresponding to 59 families. Analysis of the *T. vaginalis* degradome has revealed an expansion of protease genes, comprising approximately 400 genes identified that encode proteins of various catalytic types (aspartic, cysteine, glutamic, metallo, asparagine, serine, threonine, and another protease) [13,14]. Experimentally, a series of cysteine proteases (CPs) with molecular weights of nearly 30 kDa have been identified as virulence factors [15].

The multifactorial mechanism of pathogenesis involved in *T. vaginalis* infection is regulated by microenvironmental variations in the HUT. Fluctuations in the concentrations of iron, zinc, cadmium and polyamines, as well as the direct interaction with the epithelial host cell, among other factors, modulate the expression of virulence factor genes involved in cell adhesion, cytotoxicity, phagocytosis, hemolysis, apoptosis induction and immune evasion [16,17,18,19]. In brief, *T. vaginalis* requires adhesion to prostatic or vaginal epithelial cells and degrading extracellular matrix (EM) proteins to promote HUT infection. The *T. vaginalis* trophozoites are divided by binary fission, undergoing a morphological transformation to an amoeboid form to maximize the cellular contact area mediated by a redistribution of molecular components of actin cytoskeleton. Five glycoproteins known as adhesins—AP120, AP65, AP51, AP33 and AP23—as well as other protein factors, participate in the host cell cytoadherence process and their transcription is regulated by iron concentrations due to the presence of metal-responsive elements (MREs) located in their promoter regions [14,20,21]. *T. vaginalis* triggers host epithelial cell apoptosis by TvCP2, TvCP3, TvCP4 and CPT proteolytic activity. Others pathogenic mechanisms involve the phagocytosis process of erythrocytes and epithelial cells, among others, to acquire vital nutrients like iron and lipids, and immune evasion, in which the parasite degrades immunoglobulins and complement components using TvCP39 and TvCP65 [16,22,23,24,25,26]. Upon adhesion, *T. vaginalis* secretes virulence factors to induce cell cytotoxicity to destroy host cells. These include glycoproteins (CDF), the phospholipase A2, trichoporines, and CPs like TvCP12, TvCP39 (which degrade Collagen I, III and IV, fibronectin, hemoglobin and A and G immunoglobulins) and TvCP65 [16,22,25,26]. Recent studies have shown that the presence of Zn^2+^ upregulates the expression of several *T. vaginalis* genes implicated in important functions during pathogenesis, such as *ap65*, *tvcp39*, *tvznf1*, *mp50*, *tvmt-I*, and *tvfim-1* [27].

Metalloproteinases (MPs) degrade a wide range of substrates involved in morphogenesis, processing of normal peptides, release of cytokines and growth factors, and cell adhesion and migration, among others [28]. These hydrolases cleave peptide bonds through the action of a water molecule activated by the binding of divalent metal ions. Most metalloproteinases are characterized by the presence of a Zn^2+^ ion at their catalytic center [28,29]. Two high-molecular-weight metalloproteinases (220 to 140 kDa) were identified in *T. vaginalis*. A 47 kDa hydrogenosomal processing peptidase β-subunit (β-HPP) was characterized as a recombinant protein. Its activity was positively mediated by MnCl_2_ and strongly decreased by EDTA [30]. The highly conserved GP63 membrane metalloproteinases family, involved in parasite–epithelial cell interactions, comprises 48 members, of which 37 present predicted transmembrane domains in *T. vaginalis*. A specific member, known as TvGP63, is highly expressed on parasite membranes and participates in the cytolytic HeLa monolayer, highlighting its role in the trichomoniasis infection process [31]. In addition, the 50 kDa metalloproteinase, TvMP50, belonging to the MG clan, is overexpressed at both mRNA and protein levels in the presence of 1.6 mM of ZnCl_2_. [32]. Both the native and the recombinant protein TvMP50r degrade collagen in gelatin zimograms and are inhibited by 10 mM EDTA [27]. Crystalline structure analysis revealed fewer salt bridges and hydrogen bonds than other dipeptidases, explaining why TvMP50 functions stably as a monomer. TvMP50 is recognized in serum from male patients with trichomoniasis, but not from female patients or healthy individuals, making it a promising molecular biomarker for male diagnosis [27,32]. Additionally, TvMP50 is a secreted protein involved in cytotoxicity mechanisms by Zn^2+^ presence [33].

*T. vaginalis* gene expression is strongly regulated through a combination of unconventional basal transcription complexes, novel *cis*-acting core promoter elements, transcription factors expressed under different environmental conditions, post-transcriptional RNA–protein networks, translational and post-translational modifications, and epigenetic modifications [16,17,34,35,36]. The current understanding of all these mechanisms is important to comprehend the development and management of trichomoniasis. Male trichomoniasis has been largely neglected; for this reason, this study aimed to determine the underlying mechanisms involved in *T. vaginalis mp50* under different Zn^2+^ conditions. To characterize this potential biomarker, we analyzed the genomic organization of the *mp50* gene and the potential regulatory elements involved in its transcriptional and mRNA stability under different Zn^2+^ conditions.

## 2. Materials and Methods

### 2.1. Parasite Cultures

*T. vaginalis* female CNCD147 and male HGMN01 isolates were grown in TYM medium (trypticase–yeast extract–maltose) at pH 6.2 and 37 °C, supplemented with 10% heat-inactivated horse serum (Gibco, Waltham, MA, USA) [37]. Trophozoites were collected in the logarithmic phase for all the experiments. The Zn^2+^ condition was supplemented with 1.6 mM of ZnCl_2_ (Sigma, St. Louis, MO, USA).

### 2.2. T. vaginalis Sequence Analysis and Primer Design

The primers and sequence analysis performed in this study were designed based on the TrichDB database (http://trichdb.org/trichdb/app/record/gene/TVAG_403460 (accessed on 30 May 2026)) [38]. The oligonucleotides were designed manually and subsequently analyzed to precisely determine their specificity, %GC content, and predicted secondary structure using the Oligoanalizer 3.0 or DNAMAN 3.0 software7 (Table 1). The sequence analyses and alignments were performed using this software. The primers BTUB9 and BTUB2 were used as controls and amplified a 112 bp transcript of *T. vaginalis β-tubulin* [39].

### 2.3. mp50 mRNA Stability Assay and RT-PCR

Total RNA from parasites grown overnight in the presence or absence of 1.6 mM ZnCl_2_ was obtained from *T. vaginalis* HGMN01; the parasites were treated with 10 μg/mL of the inductor transcriptional inhibitor Actinomycin-D, and incubated at 37 °C for 0, 30, 45 and 60 min after treatment [40]. *mp50* and *β-tubulin* mRNA levels were measured in a semi-quantitative RT-PCR assay using the same primers and condition previously reported by Quintas-Granados et al. (2013) (Table 1) [27]. The band intensity in GelRed-stained agarose gels was quantified by densitometric analysis using the ChemiDoc^®^ MP (BIO-RAD, Fremont, CA, USA) and Image Lab Version 4.0 software. The pixels corresponding to t_0_ were taken as 100% of the mRNA amount under each condition. *β-tubulin* mRNA was used as a control to normalize the expression levels of *mp50*. Assuming first-order mRNA decay, the data were fitted to the exponential decay model Y = Ae^−kt^. Relative mRNA values were natural log-transformed, and linear regression of ln (Y) versus time was used to estimate the decay constant (k), calculated as the negative value of the regression slope. The *mp50* mRNA half-life was then calculated as $t_{1/2}\;=\;ln(2)/k$. The analysis was performed independently for the control and ${Zn}^{2+}$ conditions [40]. All trials were performed twice in at least three independent trials.

### 2.4. 5′ RACE

To obtain the transcription start point (tsp) and the 5′ untranslated region (5′UTR) of *mp50* mRNA from isolates CNCD147 and HGMN01 grown with or without Zn^2+^, a 5′RLM-RACE assay was performed following the manufacturer’s instructions (Invitrogen, Carlsbad, CA, USA). In brief, total mRNA was treated with calf intestine alkaline phosphatase (CIP) and a tobacco acid pyrophosphatase (TAP) to remove free phosphates and the 5′ cap. After ligating the 5′ RACE adapter, this total RNA was used to synthetize the cDNA. Finally, using an inner gene-specific primer and a primer that hybridizes with the adapter region, the PCR was performed. The resulting products were analyzed using 2% agarose gel electrophoresis. The amplicons from the two isolates in the absence and presence of Zn^2+^ were purified and cloned into the pJET 1.2/BLUNT vector (Thermo Scientific, Waltham, MA, USA) and transformed into *Eschericha coli* JM109 ultracompetent cells (Invitrogen, Carlsbad, CA, USA), and selected bacterial clones were characterized with restriction enzymes and the selected plasmids were sequenced using T7 primer. The sequence results were analyzed using several software programs, as described below.

### 2.5. 3′ RACE

To obtain the 3′ untranslated region (3′UTR) and identify the sequence involved in *mp50* mRNA polyadenylation from isolates CNCD147 and HGMN01 grown with or without Zn^2+^, the protocol for cell culture conditions and total RNA extraction is described above. After total RNA purification, a first-strand cDNA synthesis reaction was initiated using the AP adapter primer and the original mRNA was digested with a RNase H treatment. The subsequent *mp50* fragment amplification was performed using a *mp50* inner gene-specific primer that anneals to a site located 200 bp from the stop codon, and a universal amplification primer (UAP) designed for the rapid cloning of RACE products. This assay was performed following the manufacturer’s instructions (Invitrogen, Carlsbad, CA, USA). The resultant amplicons were cloned into the pGEMT-easy vector following the manufacturer’s instructions (Promega, Madison, WI, USA). Ligation products were transformed into *E. coli* JM109 ultracompetent cells (Promega, Madison, WI, USA) and selected on LB agar plates supplemented with ampicillin (100 μg/mL). White/blue colony screening was carried out using 80 μg/mL of X-gal and 0.5 mM IPTG. The positive clones were characterized by restriction analysis and sequencing. The sequence results were analyzed using several software programs, as described below.

### 2.6. PAT-PCR

From 1 µg of total RNA from each isolate grown in presence or absence of Zn^2+^, 20 ng of oligo (dT 12–18 (5′phosphorylated), Table 1) was added in a final volume of 5 µL of DEPC water and incubated for 5 min (min) at 80 °C. This mixture was incubated at 4 °C for 10 min. Another mixture containing 3 µL of 5× Superscript H-RT reaction buffer (Superscript H-RT II Invitrogen), 2 µL MgCl_2_ (25 mM, Invitrogen, Carlsbad, CA, USA), 2 µL dNTP mix (10 mM, ThermoFisher, Waltham, MA, USA) and 7 µL of DEPC water was added to the initial tube with the total RNA and the dT-oligo 12–18. The mix reaction was incubated for 5 min at 25 °C and 1 µL of Superscript-II enzyme was incubated at 42 °C for 1.5 h. After that, the samples were incubated at 70 °C for 15 min to inactivate the reverse transcriptase enzyme and treated with 1 µL of RNase H (10 mg/mL, ThermoFisher, Waltham, MA, USA) for 20 min at 37 °C. The cDNA samples were quantified using a Nanodrop 2000 (ThermoFisher Scientific, Waltham, MA, USA).

A RT-PCR was done using 1 µg of each cDNA, 5 µL of PCR Buffer 10X, 5 µL MgCl_2_ (25 mM), 1 µL of 10 mM dNTP mix, 1 µL gene-specific primer (10 µM) (Table 1), 1 µL oligo DT-anchor (10 µM), 1 µL of Taq Platinum Polymerase (Invitrogen, Carlsbad, CA, USA) and 36.5 µL of injectable water. The samples were analyzed in agarose electrophoresis gel (2%) and documented with the Chemidoc MP Gel Imaging System (BioRad, Hercules, CA, USA). As a control, specific primers were used to amplify the constitutive *β-tubuline* mRNA. We calculated the poly (A) tail length by subtracting 265 nt (corresponding to 180 nt of the coding region, 55 nt of 3′UTR, and 30 nt of the primer anchor) from the value shown in the PAT-PCR profile.

### 2.7. Cloning and Sequencing of PAT-PCR Fragments

The amplicons from PAT-PCR were cut and purified with the QIAquick PCR Purification Kit (Qiagen, Hilden, Germany) and cloned in the pJET 1.2/BLUNT vector following the manufacturer’s instructions. Plasmids were extracted with QIAGEN Plasmid Kits for Plasmid DNA Extraction (Qiagen, Hilden, Germany), sequenced with a BigDye Terminator v3. 1 Cycle Sequencing Kit and analyzed using a 3130 Genetic Analyzer (Life Technologies, Foster City, CA, USA). Sequences were analyzed using the Bioedit software 7.2 Download (Free).

### 2.8. Sequence Analysis

The *mp50* ESTs were obtained from the TrichDB database (http://trichdb.org/trichdb/app/record/gene/TVAG_403460 (accessed on 30 May 2026)) using the access number TVAG_403460 by Blast. The sequence alignment was performed in Seaview software 5.0.5 with the Clustal W algorithm. A prediction model of the secondary structure of the 3′UTR of *mp50* mRNA was created in the server Mfold http://rna.tbi.univie.ac.at/cgi-bin/RNAWebSuite/RNAfold.cgi (accessed on 26 August 2016).

## 3. Results

### 3.1. mp50 Genomic Organization

*T. vaginalis* basal transcription has a highly conserved metazoan-like initiator element (Inr) with the consensus sequence T/CA_(+1)_PyT/A; transcription initiation reveals a central adenosine residue as the transcription start site (*tsp*) [41,42]. Genomic mapping reveals that approximately 75% of protein-coding genes contain this functional core promoter element [13,43]. The remaining 25% of the massive transcriptome uses alternative sequence motifs (Motif 1, Motif 2, Motif 3, Motif 4 and Motif 5). Mutation analysis and luciferase reporter assays have confirmed that Motif 1, Motif 3 and Motif 5 are heavily involved in basal and specialized transcription [43]. Iron fluctuates during the menstrual cycle, transitioning from a highly restricted, low-iron condition to iron-rich environments during menstruation [15] *T. vaginalis* regulates its virulence factors depending on iron conditions in women. The transcriptional response and the motif sequence involved in Zn^2+^ regulation expression genes in males are not yet fully understood [27,32,33].

*T. vaginalis* possesses a distinct and functional post-transcriptional mechanism for 3′-end pre-mRNA processing and polyadenylation. Unlike metazoans that present a consensus hexanucleotide AAUAAA, *T. vaginalis* utilizes a shorter core sequence to signal polyadenylation [44]. The polyadenylation signal (PAS), the tetranucleotide UAAA, is frequently coupled to or overlaps with the UAA translational stop codon [44,45]. A mutation in PAS sequence disrupts the proper processing of the 3′ end. Relocating this tetranucleotide is enough to redirect the polyadenylation site [45]. The cleavage site (CS), defined by the novel consensus motif AA_(0–3)_UU, is located 11 to 30 nucleotides downstream of the PAS sequence. Situated up to 30 nucleotides downstream of CS, a uridine-rich element (DSE) is crucial for proper transcript maturation, resembling downstream components in higher eukaryotes [44,45]. Recent studies demonstrate that *T. vaginalis* utilizes tandem alternative polyadenylation to regulate gene expression post-transcriptionally [46].

**Figure 1:**
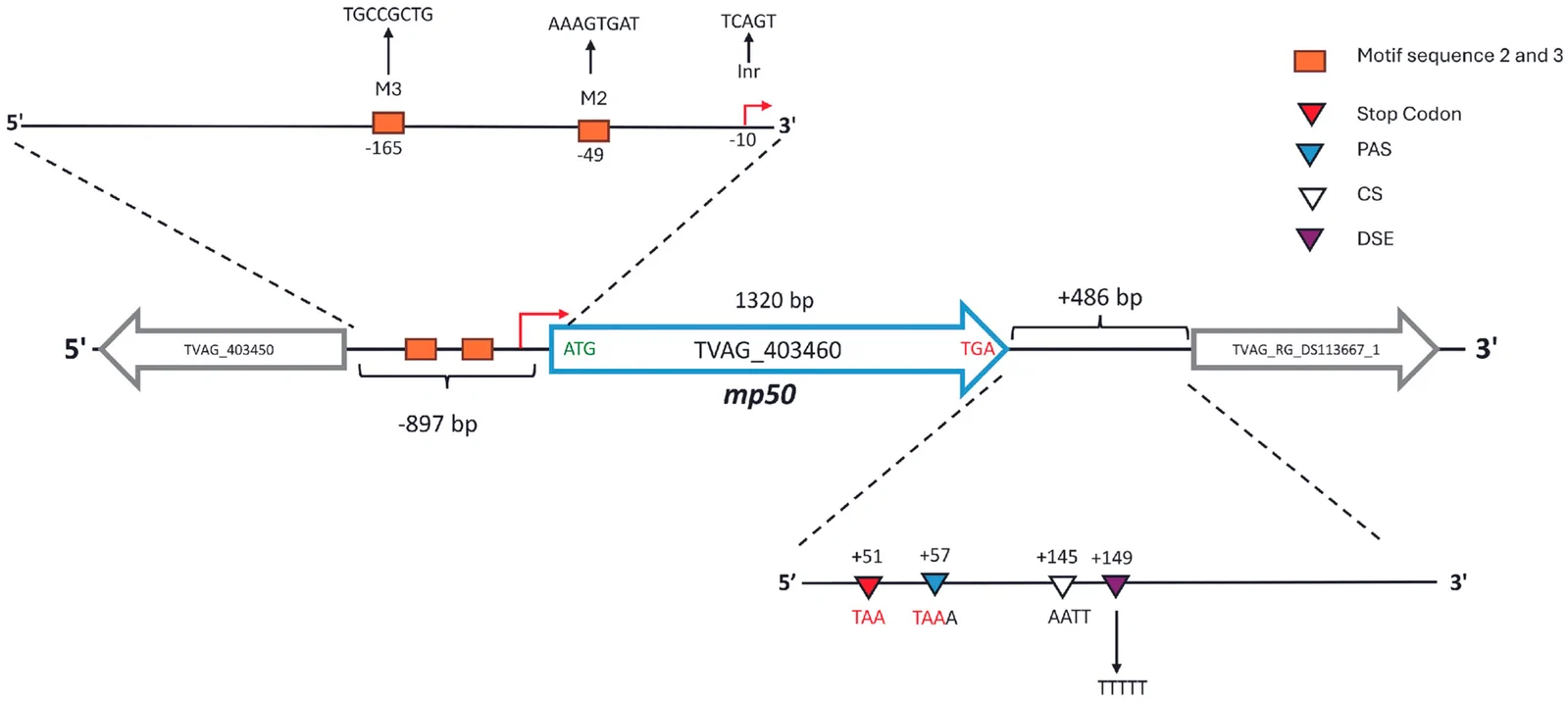
Genomic organization of the *mp50* gene (TVAG_403460) of *Trichomonas vaginalis*. The adjacent genes, the direction of transcription, and the relative distances between genomic elements are shown. In the upstream promoter region, the conserved motifs M2 and M3 are indicated, as well as the transcription start site (*tsp*), located inside the initiator motif (Inr). In the 3′ region, the termination codon (TGA), the polyadenylation signal (PAS), the cleavage site (CS), the downstream element (DSE), and the consensus sequences associated with 3′ mRNA processing are shown.

The *mp50* transcript expression was previously evaluated in two different *T. vaginalis* isolates in the absence or presence of 1.6 mM of ZnCl_2_. We previously demonstrated that *mp50* mRNA transcript increased in parasites grown in 1.6 mM of ZnCl_2_ in both *T. vaginalis* isolates [27]. To identify the mechanism involved in *mp50* mRNA expression under Zn^2+^ conditions, we first analyzed the genomic organization to determine in silico the presence of regulatory control elements located in upstream and downstream sequences of the *mp50* gene (Figure 1). *T. vaginalis mp50*, identified as TVAG_403460, is located on contig DS113667 [38]. This metalloproteinase gene (1320 bp) is flanked by a putative calcium-dependent lipid-binding protein-coding gene located 897 bp upstream of *mp50*, and a conserved hypothetical protein-coding *gene* 486 bp downstream of the *mp50* translational stop codon.

The in silico analysis of the *mp50* gene surrounding sequence reveals a canonical Inr (TCATG) located 10 bp upstream of initiation translation codon ATG and two other transcription motif sequences previously reported by Smith and colleagues (2011) [42,43]. This upstream sequence contains the proposed Motif 2 (AAAGTGAT) and Motif 3 (TGCCGCTG) (Figure 1). This gene has the translational stop codon TGA. In downstream sequences we can observe an additional stop codon (TAA) and the PAS located 51 bp downstream of TGA. The CS and DSE are present in this sequence as in previously reported sequences and taking into account the canonical PAS.

### 3.2. mp50 mRNA Stability Varies Depending on Zn^2+^ Availability

**Figure 2:**
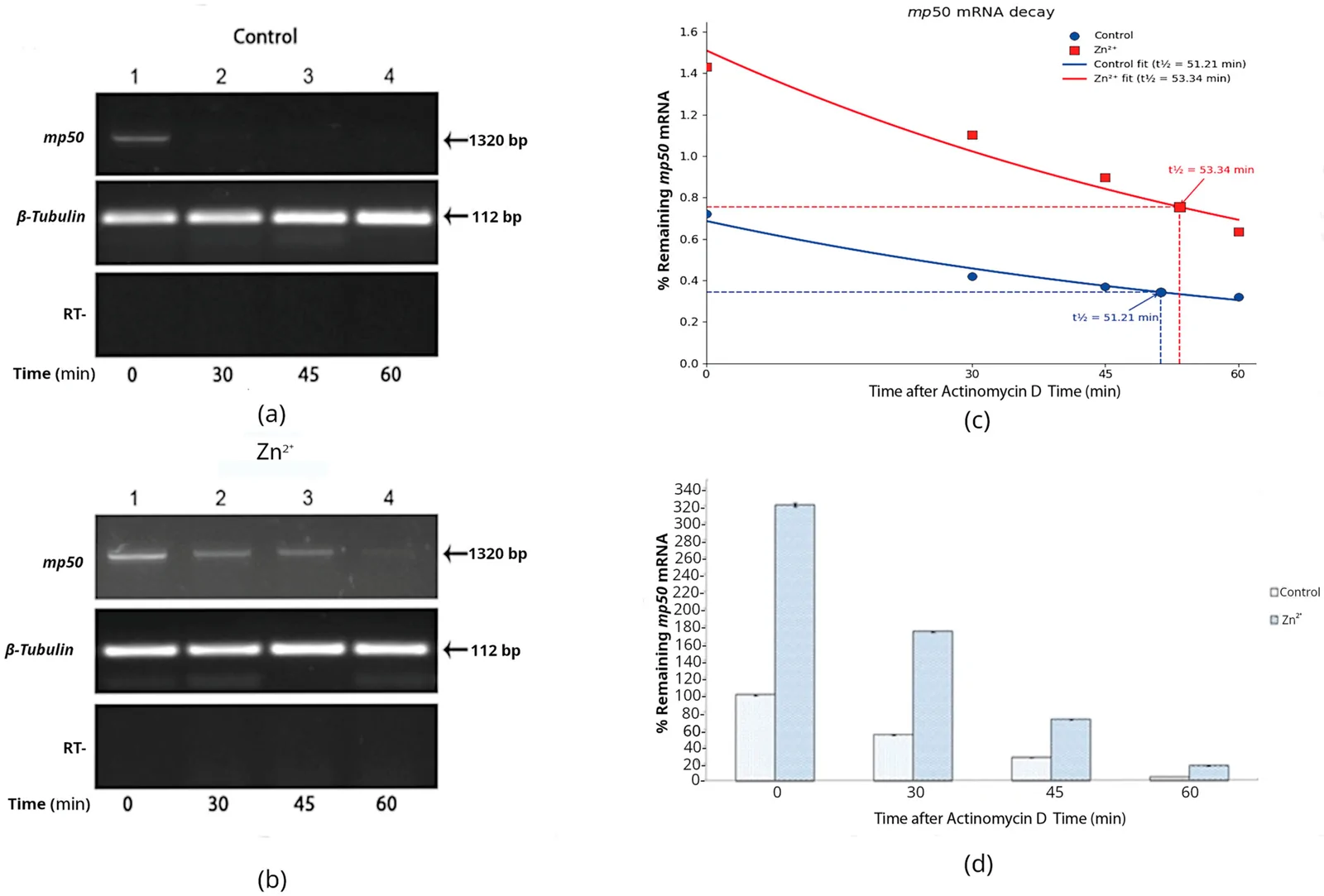
*mp50* mRNA stability in different Zn^2+^ conditions. *mp50* mRNA expression profile obtained from parasite cultures in absence (**a**) or presence (**b**) of Zn^2+^, treated with Actinomycin D, at different periods of time. For each condition, 10 × 10^6^ parasites were taken at 0 (lane 1), 30 (lane 2), 45 (lane 3), and 60 (lane 4) minutes. cDNA was synthesized and RT-PCR results are shown for both conditions (**a**,**b**). *mp50* amplicons: 1320 bp, *β-tubulin* amplicon: 112 bp. Densitometric analysis of the *mp50* and *B-tubulin* stability profile of *T. vaginalis* HGMN01 isolate are shown in panels (**c**,**d**). (**c**) Densitometric values normalized and plotted. The graph shows the decay of mRNA amount over time under both Zn^2+^ conditions. Blue line: control, red line: Zn^2+^ condition, green line: without Zn^2+^. (**d**) Densitometric relative intensity values measured using the ImageLab software 4.0 (BIORAD). Standard errors are indicated on the bars, calculated from duplicate measurements from three independent experiments.

As we previously mentioned, the *mp50* transcript expression is differentially regulated in different Zn^2+^ conditions [27,32]. We carried out an Actinomycin assay to determine whether the half-life of *mp50* mRNA differs depending on Zn^2+^ concentration. *T. vaginalis* HGMN01 was incubated with Actinomycin D in absence or presence of 1.6 mM ZnCl_2_ for different periods of time and *mp50* mRNA expression profiles were obtained by RT-PCR assays (Figure 2). In absence of Zn^2+^ the specific amplification on *mp50* transcript is found at time 0 (t_0_) after Actinomycin D addition; however, at 30, 45 and 60 min the *mp50* amplicon could not be detected (Figure 2a,b). On the other hand, the expression profile of *mp50* in the presence of Zn^2+^ showed the amplified product at 0, 30 and 45 min after Actinomycin D treatment, disappearing only at the 60 min mark. For both conditions, *mp50* transcript expression was normalized against the *β-tubulin* gene as an internal control, indicating that its transcription remained unaffected by Actinomycin treatment (Figure 2a,b). The mRNA half-life was determined by fitting the densitometric data to the exponential decay model described in the Materials and Methods (Figure 2c,d). Based on this analysis, the estimated *mp50* mRNA half-life was 51.21 min and 53.34 min for absence and presence of Zn^2+^, respectively.

### 3.3. Determination of mp50 Transcription Start Point (tsp)

To characterize the *mp50* upstream region between both isolates and determine the *tsp* in the two Zn^2+^ conditions, we amplified, cloned into the pJET-1.2/BLUNT vector, and sequenced 987 bp corresponding to the 5′ intergenic region (Figure 1). This sequence includes around 900–987 bp of a hypothetical gene located upstream of *mp50*. An alignment of the sequenced 5′ promoter region from CNCD147 and HGMN01 *T. vaginalis* isolates was compared with the *mp50* from G3 isolate reported in the TrichDB database. This analysis reveals high conservation, with 99% sequence identity. However, three main sequence variations were identified: a substitution at position −592, consecutive changes at positions −669 and −670, and an indel at position −870 in CNCD147 and HGM01 isolates (Supplementary Figure S1).

Additionally, the 5′ UTR of *mp50* mRNA was amplified by 5’RACE assay from parasites of both isolates grown with and without 1.6 mM Zn^2+^. These fragments were cloned into the pJET 1.2/BLUNT vector and sequenced, revealing that the 5 ′UTR for the *mp50* mRNA consists of approximately 10 bp relative to the ATG start codon under both experimental conditions (Figure 1, Table 2). These results indicate that the transcription initiates in the same nucleotide independently of Zn^2+^ condition or isolates.

### 3.4. Analysis of mp50 Downstream Sequence

The presence of elements in the 3′ downstream region in *T. vaginalis* has been described, modulated by iron in the *tvcp4* and *tvcp12* transcripts [26,47,48]. These genes have an IRE/IRP regulation mechanism mediated by the hairpin-loop elements in the 5′ UTR and 3′UTR, where the IREs are responsible for the negative or positive translational regulation [47,49]. Additionally, polyamines affect the *tvcp39* mRNA stability through a *cis* ERE (stem-loop) localized in the 3′ UTR that interacts with the TveIF-5A protein [24]. The *cis-acting* DNA/RNA sequence motifs are critical determinants in the mRNA regulation and its stability. *cis* sequence elements in the 3′ UTR are necessary for maturation of mRNA in *T. vaginalis* and other eukaryotes. These include the polyadenylation signal mentioned above [45].

**Figure 3:**
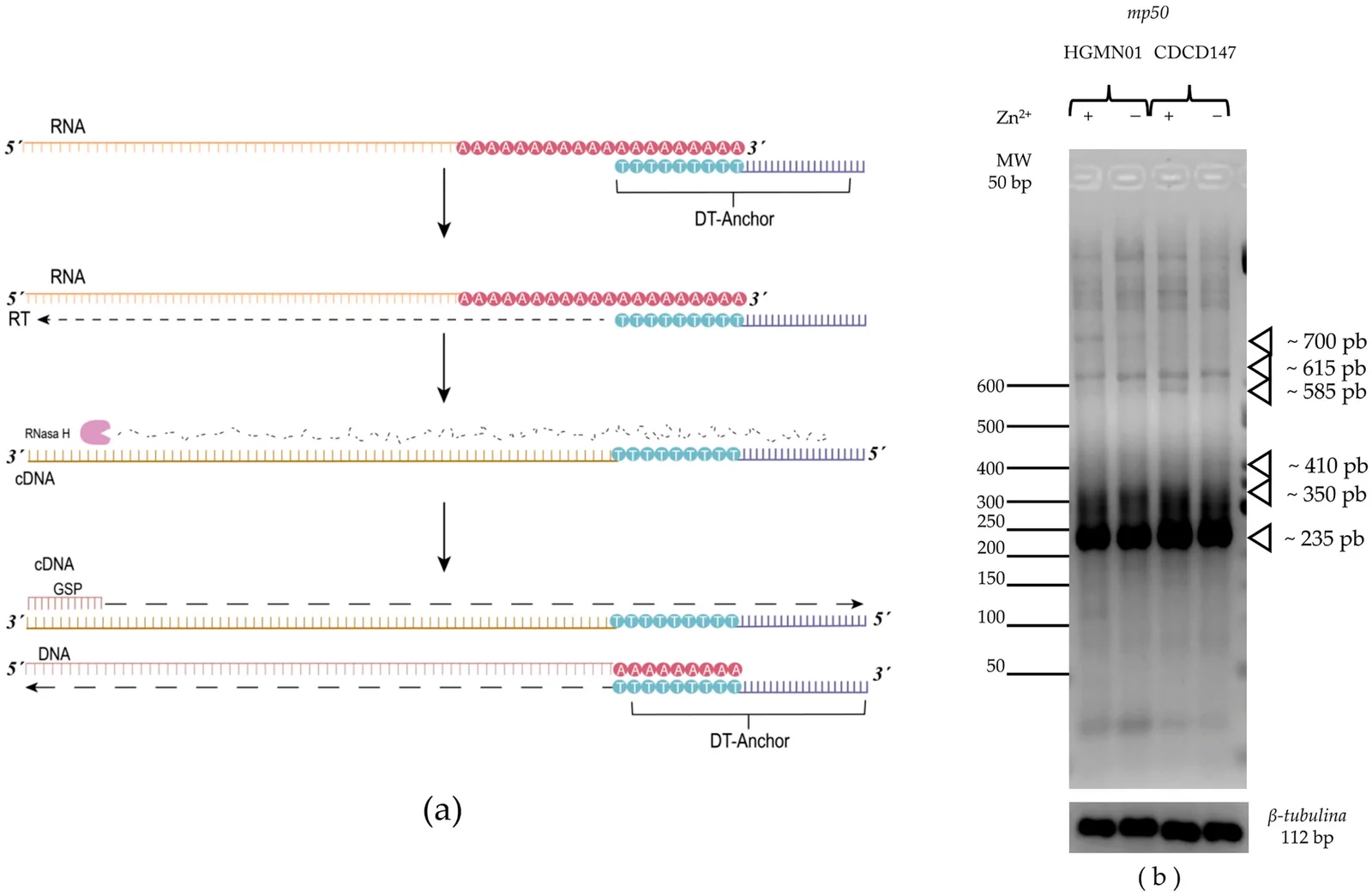
PAT-PCR protocol used to determine the poly (A) tail length of *mp50* mRNA from *T. vaginalis* male isolate HGMN01 and female isolate CNCD147. (**a**). Complete PAT-PCR protocol. (**b**). Agarose gel with PAT-PCR reactions from HGMN01 or CNCD147 *T. vaginalis* isolate grown in presence or absence of 1.6 mM Zn^2+^. MW: 50 bp molecular weight (Invitrogen). Representative image from three independent experiments.

To determine whether the length of poly (A) tract tail is one of the factors involved in the stability of *mp50* transcript, we performed PAT-PCR assays in different Zn^2+^ conditions in both isolates. The PAT-PCR complete protocol is explained in Figure 3a. The length of the poly-A tract was greater in samples of parasites grown in the presence of Zn^2+^, suggesting the involvement of a possible post-transcriptional mechanism involving mRNA stability for *mp50* (Figure 3b). The results showed a poly (A) tail length between ~235 and ~615 bp for the CNCD 147 isolate. A specific poly (A) tract had ~585 nt for CNCD147 isolate parasites. The parasite grown in the presence of Zn^2+^ showed a higher intensity band of ~235 n poly (A) tail length. In the case of HGMN01 isolate, the results showed poly (A) tail length ranging from ~235 nt to ~700 nt; interestingly, an abundant band corresponding to the poly (A) tail length of parasites grown in the presence of Zn^2+^ was ~700 nt. These results reveal differences in the poly (A) tail lengths between Zn^2+^ conditions and isolates, which may directly influence the half-life of *T. vaginalis mp50* mRNA.

**Figure 4:**
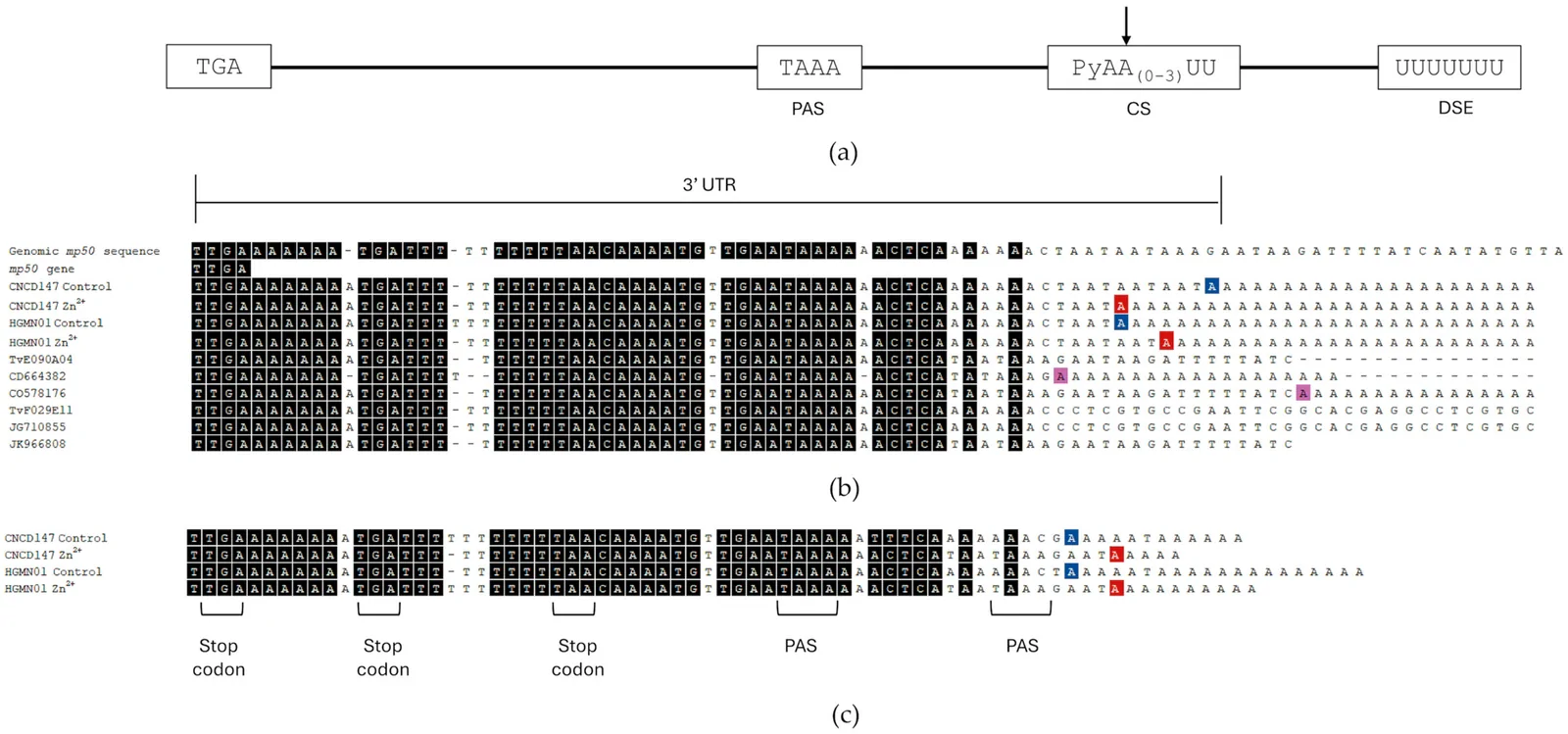
Determination of the signal and the cleavage and polyadenylation site in the 3′UTR of *T. vaginalis mp50*. (**a**) The 3′UTR sequence of the *mp50* mRNA. Modified from Espinosa and colleagues. (**b**) Alignment of the 3′UTR sequences from CNCD147, HGMN01, and *mp50* ESTs. The locations of the stop codon (TGA), the polyadenylation signal (PAS), and possible cleavage and polyadenylation sites are marked. (**c**) Experimental cleavage sites are highlighted; blue box: control condition, red box: parasites grown in Zn^2+^, pink box: CS in other growth condition. The image shows the spot codon location and the polyadenylation signal (PAS).

This method directly reveals the length of the poly (A) tail, but it does not provide information about changes in the PAS and CS regions involved in *mp50* mRNA maturation and stability. To identify the PAS, CS and DSE regions differentially recognized by the parasite polyadenylation machinery, used differentially in parasites from the two isolates, grown at different Zn^2+^ concentrations, we performed an in silico analysis of the Expressed Sequences Tags (ESTs) reported in the TrichDB database (Supplementary Tables S1 and S2, Figure 4b) [38], cloned and sequenced the DNA bands produced by PAT-PCR (Figure 4b), and performed a 3′ RACE assay (Figure 4c).

The TrichDB database contains 13 ESTs corresponding to *T. vaginalis mp50* (Supplementary Table S1) [38]. An initial alignment revealed that not all of them contained sequences extending downstream of the stop codon (TGA). ESTs with sequences that enabled the analysis of potential regulatory sequences involved in the polyadenylation of the gene were selected (Supplementary Table S2).

In silico analysis identified the PAS bases downstream of the TGA stop codon. Downstream, two distinct cleavage and polyadenylation sites were identified. Comparative alignment with five unique, non-redundant ESTs retrieved from TrichDB representing trophozoite (CD664382, 1069 nt) and amoeboid (JG710855, 564 nt) morphologies confirmed a conserved thymine (T)-rich element immediately downstream of the stop codon alongside an adenine (A)-rich element located 34 nt downstream. While the T-rich region exhibited sequence length variability, the A-rich motif consistently retained six adenines, except for EST CD664382, which exhibited a single adenine deletion (Figure 4a,b).

The DNA bands obtained in the PAT-PCR assay were cloned into the pJET 1.2/BLUNT vector, and the plasmid DNA was sequenced to evaluate the polyadenylation signals in the *mp50* transcripts of *T. vaginalis* CNCD147 and HGMN01 isolates under control conditions and Zn^2+^ stress (Figure 4b). Sequencing the 3′ UTR revealed highly conserved lengths; the CNCD147 3′ UTR maintained 55 nt across both conditions, whereas the HGMN01 3′ UTR spanned 56 nt in the control and 55 nt in the presence of Zn^2+^. The 3′RACE result reveals a similar situation (Figure 4b).

Our results suggest that *mp50* overexpression in *T. vaginalis* grown in Zn^2+^ presence might be mediated by *cis elements* such as the poly (A) tract or regulatory elements at the 3′UTR of mRNA. Analysis of ESTs, sequencing of *mp50* transcripts obtained via PAT-PCR, and 3′RACE assays suggest the use of multiple PA sites and CS.

**Figure 5:**
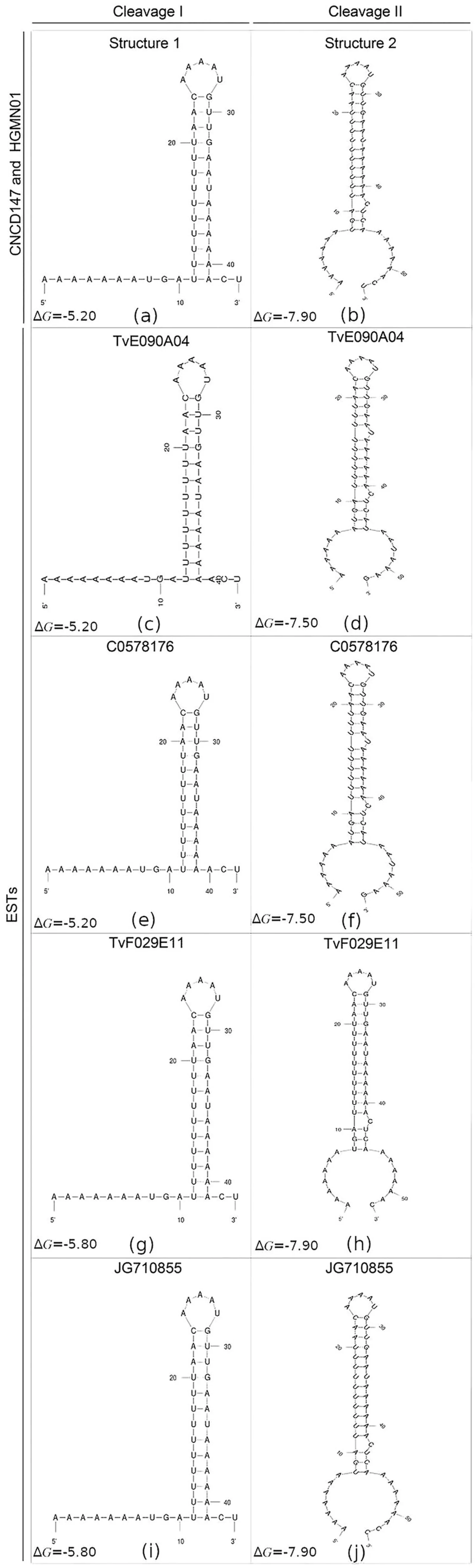
Determination of the secondary structure of the *mp50* mRNA from isolates CNCD147 and HGMN01 and EST sequences. The models included CS I (**a**) and CS II (**b**), referred to as the CNCD147 and HGMN01 structures. For the EST sequences, both cleavage and polyadenylation sites were also considered (**c**–**j**). The structures correspond to the sequence with cleavage and polyadenylation sites. (**a**) CNCD147 and HGMN01 (structure 1). (**b**) Modeling of the CNCD147 and HGMN01 sequences at cleavage and polyadenylation site II (structure 2). For the ESTs: (**c**) TvE090A04 CS I. (**d**) TvE090A04 CS II. (**e**) C0578176 CS I. (**f**) C0578176 CS II. (**g**) TvF029E11 CS I. (**h**) TvF029E11 CS II. (**i**) JG710855 CS I. (**j**) JG710855 CS II. Cleavage site I: CS I; cleavage site II: CS II. AG is indicated in each step-loop.

Then, the two cleavage sites were used to model the secondary structure of the *mp50* 3′UTR mRNA region of both isolates and the *mp50* EST sequences. The first structure using cleavage site I (CS I) showed a loop of 7 nt and a stem of 24 nt, and an atypical U-G base-pairing in position 20 with a dG of −5.20 (Figure 5a). In the case of ESTs Tv090A04 and C0578176, the analysis showed a loop of 7 nt, an atypical stem of 22 nt, and a base-pairing of U-G in 19 positions with dG −5.20 (Figure 5c,e). Then, the models corresponding to CS I of ESTs TvF029E11 and JG710855 showed a loop of 7 nt and a stem of 24 nt, and the atypical base-pairing of U-G in 20 positions in both structures (Figure 5g,i).

The cleavage model for CNCD 147 II showed a secondary structure model with a loop of 7 nt, a stem of 30 nt and an atypical base-pairing of U-G in 20 positions. In addition, a loop with a cytosine at position 42 was observed (Figure 5b). The models of cleavage site II (CS II) for CNCD147 showed a loop of 7 nt and a stem of 30 nt with the same atypical base-pairing of U-G at position 19; in addition, it showed a bulge with a C at position 41, and the structure had a dG of −7.50 (Figure 5b). The ESTs of Tv090A04 and C0578176 showed a loop of 7 nt and stem of 34 nt with the same atypical U-G base-pairing at position 19. In addition, they showed two bulges at positions 34 and 41 respectively; this structure had a dG of −7.50 (Figure 5d,f). The ESTs of TvF029E11 and JG710855 showed a loop of 7 nt and stem of 34 nt with the same atypical U-G base-pairing in position 20; in addition, they had a bulge in position 41. This structure had a dG of −7.90 (Figure 5h,j).

## 4. Discussion

The regulation of the expression of mRNA in *T. vaginalis* has been studied at the transcriptional and post-transcriptional level in the presence of polyamines and with Fe^3+^ as stimulus [16,50]. In the male environment, the presence of a high Zn^2+^ concentration is the first barrier of the immune system against microorganisms in the urogenital tract; however, *T. vaginalis* is capable of surviving these urogenital tract adverse conditions [8,9] In this experimental research, we used *mp50* as a model to analyze mRNA stability regulation in the absence and presence of Zn^2+^. This analysis indicates that *mp50* transcription and post-transcriptional regulation mediated by Zn^2+^ could differ from canonical or previously reported iron regulation mechanisms.

Transcription, one of the first events, is a process to generate a copy of RNA from DNA. Specific stimuli affect the transcription positively or negatively, activating or repressing the binding of transcription factors to promoter regions or *cis* elements located in the upstream region of a gene, to produce a specific mRNA. The mature mRNA has regions known as untranslated regions (UTRs) at the 5′ and 3′ ends, where gene expression regulatory elements are located, such as promoters and MRE sequence elements, stem-loop structures or specific sequence that are recognized by RNA-binding, and the poly (A) tail. Identifying these elements is important to understand the parasite pathogenicity, the virulence factor expression and biomarker characterization. Previous results indicate differential expression of *mp50* mRNA (and hence of the TvMP50 protein) in a Zn^2+^-concentration-dependent manner. This led us to evaluate the factors that might be influencing this differential expression at a transcriptional and post-transcriptional level.

A short half-life allows mRNA to be present for a minimum amount of time for translation into proteins to take place. The intrinsically short or long half-life does not necessarily mean that regulation is dependent on mRNA stability, as there is also an increase in half-life when mRNA synthesis is reduced or repressed [51]. The Actinomycin D assay results demonstrated that the *mp50* mRNA half-life varies depending on Zn^2+^ concentration. Previously, we reported differences in mRNA quantity in parasite growth with or without Zn^2+^ between both isolates (Figure 2) [32]. This effect could be attributed to changes in *tsp*, the use of alternative Inr or another sequence located in the upstream region of the *mp50* gene. As for the downstream sequence of *mp50*, the mRNA stability could be affected by the poly (A) tail length [51,52,53], the alternative use of PAS sequences [46], and/or the generation of different stem-loop structures that modify mRNA stability, as occurs in the IRE/IRP system in *T. vaginalis* [16,40,49]. The half-life of mRNA could be influenced by different processing events in the 5′UTR and 3′UTR. In the 5′ upstream region, an mRNA may present several *tsps*, which directly results in varying sizes of 5′UTR. In silico analysis of the *mp50* gene upstream region revealed the presence of different motifs that could generate alternative *tsps* (Figure 1). These results do not exclude the possibility that other upstream factors, Motif 2 or 3, may be involved in transcription regulation mediated by Zn^2+^. Further studies are required to determine the function of these sequence motifs. A functional analysis of these sequence elements is necessary to understand and characterize the sequence control elements implied in transcription initiation mediated by Zn^+2^ concentrations.

Another important aspect that could modulate the mRNA half-life is the polyadenylation, a post-transcriptional process that takes place predominantly in the cellular nucleus where a poly (A) polymerase adds adenine (A) residues to the 3′ end. The poly (A) tail is necessary to transport mRNAs from the nucleus to the cytoplasm and increases transcript stability, mRNA decay and translation efficiency [54,55]. The poly (A) tail can be up 200–250 nt in mammals and 90 nt long in yeast [56,57,58]. The nascent pre-mRNA is polyadenylated in two steps: cleavage and polyadenylation. The enzymatic machinery involved in theses process is well characterized and composed of approximately 20 individual proteins [59]. Several sequence motifs located in the 3′ end, previously described in eukaryotes, are involved in these two processes. The first step is undertaken by the endoribonuclease CPSF73 [60,61,62]. This protein recognizes the CS (a CA dinucleotide in mammals) located between the PAS—with the AAUAAA hexamer, predominately located 21 or 22 nt upstream of CS—and DSE with a GU-rich tract [59,63,64]. The poly (A)-binding proteins PAP, PABP and the nuclear isoform PABPN1 are essential for the poly-A polymerization [62,65,66]. Deadenylation contributes to mRNA degradation and these two processes are carried out by highly regulated multiprotein sub-complexes acting in a coordinated manner [55].

We obtained the poly (A) tail length from the *mp50* mRNA in both conditions (absence and presence of Zn^2+^) from two isolates (CNCD1457 and HGMN01) (Figure 3 and Figure 4). Our results show that in presence of Zn^2+^ the tract of polyadenines is longer, which could be associated with the presence of the cation. Interestingly, the differences in poly (A) tail lengths suggest a possible regulation mechanism at the translation level consistent with the overexpression of TvMP50 protein in the presence of Zn^2+^ (Figure 3). In protozoans, studies have previously suggested the role of the 3′UTR of mRNA in translational regulation for many species [67]. In addition, it has been demonstrated that the 3′UTR influences the rates of turnover of mRNAs like the *hsp83* gene in *L. donovani* and it mediates the metabolism of glucose as the expression of the procyclin and glucose transporter in *T. brucei* [68]. During oogenesis and in early development in metazoans, a predominant form of translational regulation is a cytoplasmic change in the length of the poly (A) tail [69].

In contrast with our results, no differences were found in the length of poly (A) tail for the *gapdh* gene in samples of three different protozoa (*L. donovani*, *T. brucei*, and *T. vaginalis*), where the PAT-PCR technique was used to measure the poly (A) tail, although it has been demonstrated that this technique is capable of detecting differences until 10 nt [70].

Via PAT-PCR, we obtained the poly (A) length and 3′UTR sequence of two isolates in two different conditions, Zn^2+^ presence and absence. We did find differences in the 3′UTR length of both isolates (Figure 3b). The in silico analysis showed that the 3′UTR has two possible cleavage and polyadenylation sites, but interestingly the analysis shows the differences in the poly (A) tail length and the polyadenylation signal (Figure 1). These results are not surprising since other studies have reported mediation of translation control mechanism by poly (A) tail length.

The poly (A) tail length has been described as involved in mRNA stability, and short tails promote mRNA degradation. Currently, studies have demonstrated that changes in the size of poly (A) may be involved in translational control. Different sizes of poly (A) tail in the mRNA are involved in the progressivity of poly (A) polymerase and other factors could determine the translation efficiency such as PABPC protein [52,71].

The *cis* elements of the 3′ UTR are involved in the post-transcriptional regulation mechanism; we found two possible cleavage poly (A) sites and a possible stem-loop element in the *mp50* 3′UTR (Figure 5). *T. vaginalis* has been described as a post-transcriptional mechanism mediated by an IRE/IRP system, which involves a stem-loop structure in the 5′ or 3′UTR which regulates the translation of the protein. This mechanism was described for *tvcp12* and *tvcp4* genes. The loops of these cis elements contain the sequences GGCACA (*tvcp4)* and UAAUUG (*tvcp12*) respectively and also an atypical IRE structure not conserved in other organisms [16,48,49]. These elements are recognized by IRPs, which bind to IRE, which is mediated by Fe^3+^ affecting the stability or translation of the mRNA [48].

Currently, is important to know how the length of the poly (A) tail stimulates the translation process. One possibility might be that the cytoplasmic 3′ poly (A) tail induces 5′ cap ribose methylation which produces translational activation [72]. Another possibility is that the poly (A) tail recruits many proteins of the PABPC family that recognize the poly (A) tail in the mRNAs, which increases the interaction with eIF4G/eIF4F; this complex recognizes the cap, promotes the stability of the ribosome complex and produces an increase in the translation process [57,65]. The poly (a) signal and cleavage site can change depending on zinc concentrations, and it may produce the stem-loop as shown in Figure 5. The analysis of these structures indicates several AGs; however, the function of these structures should be analyzed. For this, we propose an interaction assay between the stem-loop and the cytoplasmic proteins from parasites growth under different zinc conditions and using transcripts with some mutations to confirm the specific interaction.

**Figure 6:**
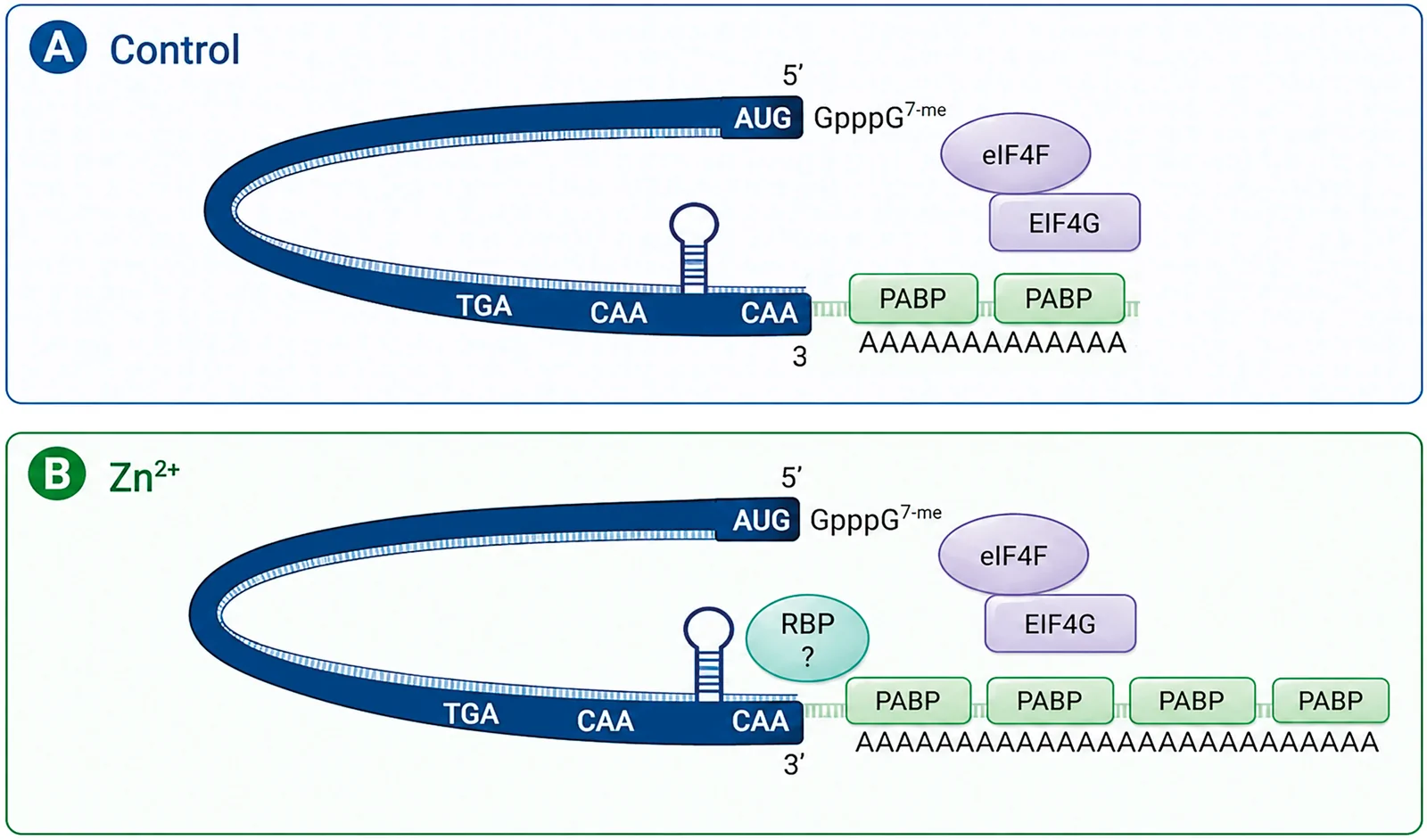
Proposed model for Zn^2+^-dependent regulation of *mp50* mRNA stability and translation. (**A**) Under control conditions, *mp50* mRNA exhibits a shorter poly (A) tail with limited PABP association. The stability and half-life of this *mp50* mRNA are reduced due to the poly (A) tail length. (**B**) In the presence of Zn^2+^, the model proposes enhanced transcript stabilization, potentially mediated by a RNA-binding protein (RBP) associated with a secondary RNA structure, along with increased binding of PABP to an extended poly (A) tail. These interactions may promote *mp50* mRNA stability and the translation initiation through eIF4E and eIF4G interactions.

In the presence of Zn^2+^, PABPC binds to the poly (A) tail and allows PABP to connect to the longer tails to help ribosomes load onto the *mp50* mRNA more efficiently, and it increases the amount of TvMP50 (Figure 6). The interaction between eIF4G and PABPC causes an increase in the *mp50* translation levels (Figure 6B). Further research is required to elucidate this hypothesis.

## 5. Conclusions

Epidemiological research reveals health disparities and risk factors that contribute to the prevalence of trichomoniasis worldwide. Risk factors correlated with higher infection rates include female sex, older age, low socioeconomic status, educational degree, and the number of sexual partners within the preceding years. *T. vaginalis* shares an epidemiologic association with other sexually transmitted infections, presenting alongside bacterial vaginosis, fungal infections and co-infections with virus such as Human Papillomavirus, among others. Furthermore, 40% to 60% of women diagnosed with *T. vaginalis* suffer from concurrent bacterial vaginosis, creating a synergistic vaginal dysbiosis that can elevate the pathogenic persistence [6,73,74,75].

The relationship between male *T. vaginalis* infection and prostate cancer requires the complete characterization and development of specific biomarkers. The 50 kDa metalloproteinase immunogenic biomarker expressed during male infection requires a deep understanding of molecular mechanisms involved in *T. vaginalis* MP50 gene expression.

The poly (A) tail length was longer in *T. vaginalis* parasite cultures grown in Zn^2+^, suggesting a possible *mp50* mRNA stability mechanism induced by this cation presence. Analysis of these results suggests that the stability of this transcript depends directly on the culture conditions and is determined by a series of factors; the additive effect of PAS, CS, and the formation of the stem-loop structures determine the stability of the *mp50* mRNA. A functional study of each of these regions, using targeted mutation assays, will be necessary to precisely determine the effect of each of these regions.

These results propose alternative polyadenylation sites involved in transcript stability depending on growth conditions.

## Figures and Tables

**Table 1:** Oligonucleotides used in this study.

Primer	Sequence	Reference
BTUB9	5′-CATTGATAACGAAGCTCCTTTACGAT-3′	[39]
BTUB2	5′-GCATGTTGTGCCGGACATAACCAT-3′	[39]
5′RACE adapter	5′-GCUGAUGGCGAUGAAUGAACACUGCGUUUGCUGGCUUUGAUGAAA-3′	5′RACE kit
5′RACE outer	5′-GCTGATGGCGATGAATGAACACTG-3′	This article
5′RACE inner	5′-CGCGGATCCGAACACTGCGTTTGCTGGCTTTGATG-3′	This article
Oligo(dtT)-anchor	5′-GCGAGCTCCGCGGCCGCGT_12_-3′	3′RACE kit
AUAP	5′-GGCCACGCGTCGACTAGTAC-3′	3′RACE kit
GSP sense-mp50	5′-CGACTGGGACTGGGCTTTGAAACTCGCTGA-3′	This article
5′RACE outer-mp50	5′-CCAAGCTTTCAAAGTACTCTTGAAGC-3′	This article
5′RACE inner-mp50	5′-TTTCTCAATATAAAGTGTTGATTGTCCGGT-3′	This article
Sense	5′-GCTCCTTAGCCTCAATAACTTTAAGC-3′	This article
Antisense	5′-CACTTCGATAAGGTTCTTGCGAT-3′	This article

Primers designed for this research article.

**Table 2:** Sequences of upstream *mp50* region from *T. vaginalis* isolates CNCD147 and HGMN01 in the absence and presence of Zn^2+^.

Isolate	Upstream *mp50* Sequence	
Condition	Control	Zn^2+^
CNCD147	AAAATTTTTGATGTCAGGTGA	AAAATTTTTGATGTCAGGTGA
HGMN01	AAAATTTTTGATGTCAGGTGA	AAAATTTTTGATGTCAGGTGA

A 10 nt sequence from the 5′ UTR of the mRNA encoding the *mp50* gene (underlined) and translation start codon.

## Data Availability

The original contributions presented in this study are included in the article/supplementary material. Further inquiries can be directed to the corresponding author.

## Supplementary Materials

The following supporting information can be downloaded at https://www.mdpi.com/article/10.3390/microorganisms14081755/s1. Figure S1: Alignment of the 5′ promoter sequenced region from CNCD147 and HGMN01 *T. vaginalis* isolates and the reported sequence from *mp50* from G3 isolate reported in TrichDB database; Table S1: 13 ESTs reported in the TrichDB database (www.trichdb.org) for *mp50 Trichomonas vaginalis*; Table S2: ESTs reported in the TrichDB database (www.trichdb.org) used in silico analysis for *mp50 Trichomonas vaginalis*.

## Abbreviations

STI: Sexually Transmitted Infection; PC: Prostate Cancer; HUT: Human Urogenital Tract, CP: Cysteine Protease, MP: Metalloprotease; UTRs: Untranslated Regions; PAS: Polyadenylation Signal; CS: Cleavage Site; DSE: Downstream Sequence Element; nts: Nucleotides, bp: Base Pair; EST: Express Sequence Tag.
