# Supplementary material for: Analysis of cis Elements in the mp50 mRNA of Trichomonas vaginalis
Source: Microorganisms. 2026 Aug 10;14(8):1755. doi: 10.3390/microorganisms14081755 (PMC13515687; doi:10.3390/microorganisms14081755)
Supplement: Supplementary file 1 [file microorganisms-14-01755-s001.zip › Table S2.pdf]

Supplementary Table S2. ESTs reported in the TrichDB database (www.trichdb.org) used *in silico* analysis for *mp50 Trichomonas vaginalis*.

Accesion Number	*T.vaginalis* isolate	Growth condition	Lenght (bp)
JG710855	*T. vaginalis*	Ameboid	564
TvF029E11	*T. vaginalis* G3	Ameboid	564
JK966808	*T. vaginalis*	Trophozoites	639
CO578176	*T. vaginalis*	Trophozoites mid-log fase	676
TvE019C04	*T. vaginalis* G3	Unsynchronized culture	854
CD664382	*T. vaginalis*	Trophozoites mid-log fase	1069

The accession number reported in the database, the growth conditions under which it was obtained, and the length in bp are indicated.
